# Prevalence of *Staphylococcus* spp. from human specimens submitted to diagnostic laboratories in South Africa, 2012–2017

**DOI:** 10.4102/sajid.v38i1.477

**Published:** 2023-01-30

**Authors:** Themba T. Sigudu, James W. Oguttu, Daniel N. Qekwana

**Affiliations:** 1 Department of Agriculture and Animal Health, College of Agriculture and Environmental Sciences, University of South Africa, Pretoria, South Africa; 2 Department of Health and Society, School of Public Health, Faculty of Health Sciences, University of the Witwatersrand, Johannesburg, South Africa; 3 Department of Paraclinical Sciences, Faculty of Veterinary Science, University of Pretoria, Pretoria, South Africa

**Keywords:** *Staphylococcus* species, coagulase negative, CoNS, coagulase positive, CoPS, humans, South Africa

## Abstract

**Background:**

Although staphylococci are commensals of the skin and mucosa of humans and animals, they are also opportunistic pathogens. Some coagulase-negative *Staphylococcus* spp. (CoNS), such as *S. haemolyticus* and *S. epidermidis*, are reported to be zoonotic.

**Objectives:**

The prevalence of coagulase positive (CoPS), CoNS and coagulase-variable *Staphylococcus* spp. isolated from human clinical cases in South Africa was investigated.

**Method:**

Retrospective records of 404 217 diagnostic laboratory submissions from 2012 to 2017 were examined and analysed in terms of time, place and person.

**Results:**

Of the 32 different species identified, CoPS were the most frequently isolated (74.7%), followed by CoNS (18.9%). Just over half (51.2%) of the *Staphylococcus* isolates were from males, while females contributed 44.8%. Patients aged 0–4 years contributed the most (21.5%) isolates, with the highest number coming from KwaZulu-Natal (32.8%). Urinary specimens accounted for 29.8% of the isolates reported. There was no variation in the number of *Staphylococcus* isolates reported in the autumn (25.2%), winter (25.2%), spring (25.1%) and summer (24.5%) seasons.

**Conclusion:**

This study demonstrated the diversity of *Staphylococcus* spp. isolated from humans and the magnitude of infection, with the most predominant species being *S. aureus* and *S. epidermidis.*

**Contribution:**

Although most isolates were CoPS, the isolation of CoNS seen in this study suggests a need to improve infection control measures in a South African context. More research is needed to investigate the determinants of the observed variations in the study.

## Introduction

*Staphylococcus aureus* are natural residents on the skin and mucous membranes of a wide range of host species.^1^ For example, healthy people carry *S. aureus* as a part of their normal microbiota in the nose, throat, perineum or skin.^2,3^ However, these organisms may become pathogenic if they gain entry into the host tissue through trauma of the cutaneous barrier.^4,5^

The clinical significance of many *Staphylococcus* spp. has traditionally been dismissed because of their ubiquitous nature, and as a result, their isolation has been considered contaminants.^6^ However, this perception is changing because of many species emerging as important causes of nosocomial infections, particularly for medical devices and in immunocompromised patients.^7^ In addition, *Staphylococcus* spp. have been reported in clinical conditions such as boils, impetigo, food poisoning, cellulitis and toxic shock syndrome. However, skin infections including cellulitis, folliculitis, furuncles, impetigo and subcutaneous abscesses are the most common.^8^

*Staphylococcus* spp. are classified into coagulase positive or negative based on their ability to produce the coagulase enzyme that causes blood to clot. Coagulase-positive *Staphylococcus* (CoPS) are responsible for a variety of opportunistic infections not only in humans but also in animals.^1^ Transmission of these organisms occurs through direct contact with colonised or infected persons or animals or through indirect contact with contaminated objects. Among CoPS, *S. aureus* is the most common species. However, CoPS such as *Staphylococcus pseudintermedius* and *Staphylococcus schleiferi* subspecies *coagulants* have also been reported in clinical conditions.^9^ *Staphylococcus argenteus* and *Staphylococcus schweitzeri*, previously known as divergent *S. aureus* clonal lineages, were recently identified as novel, difficult-to-delimit *S. aureus* complex CoPS. The lack of knowledge about their epidemiology, medical significance and transmission risk makes definitive recommendations difficult. However, there is growing evidence suggesting that *S. argenteus* pathogenicity is similar to that *of S. aureus*, while no *S. schweitzeri* infections have been reported.^10^

Although coagulase-negative *Staphylococcus* (CoNS) are predominant in domestic animals, human infections have also been reported.^6^ For example, *S. epidermidis* has been reported as causing severe infections in immunosuppressed patients and those with central venous catheters.^11^ Meanwhile, *S. saprophyticus* has been implicated in genitourinary tract infections. In recent years, *S. lugdunensis* also has emerged as an important cause of disease in humans.^6^ In addition, members of ‘*S. epidermidis* group’ that include *S. capitis, S. hominis, S. simulans*, and *S. warneri have been reported in human clinical cases*.^6^

Currently, there are limited published studies investigating the distribution of *Staphylococcus* spp. among humans in South Africa. Available studies have focused mainly on *S. aureus* infections.^11,12,13^ Improved understanding of the burden of infections caused by the various *Staphylococcus* spp. in settings such as South Africa is needed. This will help increase the awareness of *Staphylococcus* infections and guiding empirical treatment. Therefore, the objective of this study was to describe the distribution of *Staphylococcus* spp. isolated from human samples submitted to diagnostic laboratories in South Africa based on person, time and space.

## Methods

### Study design and data extraction

A cross-sectional retrospective study design was implemented. Records of 404 217 *Staphylococcus* spp. isolated from 123 diagnostic laboratories countrywide during the period 2012 and 2017 were extracted from the National Health Laboratory Service (NHLS) electronic database. These laboratories service the public health sector hospitals and receive samples from all levels of healthcare service (district, regional, tertiary and central) in South Africa. Specimens submitted for microbiological analysis included skin, blood, urine, catheters (central venous catheter and haemodialysis), nasopharyngeal fluid and specimens from other body sites. For each isolate, data extracted from the NHLS database included a combination of demographic information, clinical information, antimicrobial sensitivity test results and information on the temporal trends.

Data of all *Staphylococcus* spp. identified between 2012 and 2017 were extracted from the NHLS data set. For each isolate, the following variables were extracted specimen type, age, gender, date and locality (province).

### Data management

To prepare the data for analysis, the data were inspected for inconsistencies such as missing information, incorrect addresses and duplicate entries. No duplicates were identified and no mixed infections were reported.

The variable ‘age’ was recategorised into the following 14 categories using the cohort-component method for population estimation produced by Statistics South Africa (Stats SA), 0–4 years, 5–9 years, 10–14 years, 15–19 years, 20–24 years, 25–29 years, 30–34 years, 35–39 years, 40–44 years, 45–49 years, 50–54 years, 55–59 years, 60–64 years and > 65 years (p. 25).^14^ Months were categorised into four seasons: autumn (March, April and May); winter (June, July and August); spring (September, October and November) and summer (December, January and February). The type of specimens was classified into the following five categories: skin, urinary, blood, nasopharyngeal fluid and ‘All other sites’. ‘All other sites’ included unidentified and specimen types with a percentage less than 0.02% (Online Appendix 1).

### Data analysis

All data processing and analyses were performed using Stata Statistical Software (Release 13. College Station, Texas: StataCorp LP).^15^ Crude and factor-specific proportions for categorical variables and their corresponding 95% confidence intervals (95% CI) were computed and presented based on time, person and place. Annual changes in the proportion of *Staphylococcus* spp. were displayed using temporal graphs.

### Ethical considerations

Access to the NHLS database and patient information is restricted to laboratories staff working within the NHLS and can only be accessed at the premises of the NHLS. Thus, data extraction was carried out by the NHLS staff and de-identified data were provided to the researcher. Confidentiality and anonymity were always maintained by ensuring that patient personal information was not included in articles and reports. In addition, permission to use the data was obtained from the NHLS. Ethical approval was obtained from the University of South Africa College of Agriculture & Environmental Sciences’ Health Research and Animal Research Ethics Committees (Ref: 2018/CAES/107). The data were kept safe from unauthorised access, accidental loss or destruction. Data in the form of softcopies were kept as encrypted files in computers and flash drives.

## Results

In a total of 404 217 staphylococci isolated, 32 different *Staphylococcus* spp. were identified. Coagulase-positive *Staphylococcus* comprised 74.6%, while CoNS made up 19.2%. The remaining 6.4% were *coagulase*-variable (meaning that some strains can be coagulase positive and some coagulase negative).

Among the CoPS, *S. aureus* was the most commonly isolated species (74.4%), followed by *S. intermedius* (0.1%), and then *S. pseudintermedius* (0.05%). On the other hand, among the CoNS, *S. epidermidis* (11.0%) was most commonly isolated, followed by *S. haemolyticus* (3.3%) and *S. hominis* (1.6%) (Table 1).

**TABLE 1:** Distribution of *Staphylococcus* spp. isolated at diagnostic laboratories between 2012 and 2017.

Organism	*n*	%	95% CI
**CoPS**			
*S. aureus*	300 779	74.4	73.40–73.50
*S. intermedius*	540	0.1	0.12–0.14
*S. pseudintermedius*	223	0.1	0.05–0.06
**CoNS**			
*S. epidermidis*	44 364	11.0	10.90–11.10
*S. haemolyticus*	13 406	3.3	3.30–3.40
*S. hominis*	6443	1.6	0.15–0.16
*S. saprophyticus*	4041	1.0	0.97–1.03
**CoPS/CoNS**			
Unspeciated *Staphylococcus*	25 674	6.4	6.30–6.40
*S. schleiferi*	74	0.02	0.01–0.02
*S. hyicus*	77	0.02	0.01–0.02

CI, confidence interval; CoPS, coagulase-positive *Staphylococci*; CoNS, coagulase-negative *Staphylococci*.

### Distribution of *Staphylococcus* spp. by the demographic characteristics of patients

**FIGURE 1:**
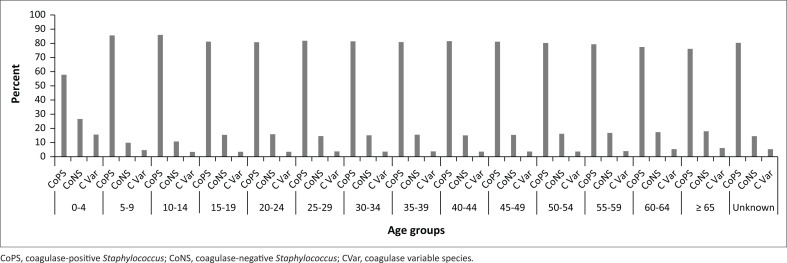
The distribution of coagulase-positive *Staphylococcus,* coagulase-negative *Staphylococcus* and *coagulase variable species* among patients by age group for the period 2012–2017.

Although overall patients aged 0–4 years contributed the highest number of *Staphylococcus* spp. (21.5%), as shown in Figure 1, in comparison to the other age groups, patients aged 0–4 years contributed the lowest number of CoPS. However, the proportion of CoNS was higher among patients aged 0–4 in comparison to other age groups. Meanwhile, there was no distinct variation in the number of the CoNs, CoPS and coagulase variable species between the age group 5–9 years and above, and unknown (patients whose age was not captured).

The highest proportion of *Staphylococcus* isolates came from KwaZulu-Natal province (32.8%). Gauteng (21.1%) contributed the second highest number followed by the Western Cape (14.9%), and Eastern Cape (13.5%).

Specimens from 81 different sources were submitted and classified into five main groups. Overall, skin was the most common specimen (39.7%), followed by urine (29.8%), blood (20.5%) and nasopharyngeal swab (9.8%). Specimens from other sites made up of 0.2% of the total specimens identified (Table 2).

**TABLE 2:** The distribution of the different groups of *Staphylococcus* spp. by gender, age, province, season, year, and specimen type, 2012–2017.

Variable	Total isolates		CoPS		CoNS		C Var	
	*n*	%	*n*	%	*n*	%	*n*	%
**Gender**								
Males	210 858	51.2	163 540	77.6	34 593	16.4	12 725	6.0
Females	181 270	44.8	136 071	75.1	32 564	17.9	12 590	7.0
Unknown	12 089	4.0	7211	59.7	3181	26.3	1697	14.0
**Season**								
Autumn	101 673	25.2	76 883	75.6	18 239	17.9	6551	6.4
Winter	101 863	25.2	77 539	76.1	17 871	17.5	6453	6.3
Spring	101 515	25.1	76 547	75.4	17 937	17.7	7031	6.9
Summer	99 166	24.5	75 053	75.7	17 501	17.7	6612	6.6
**Year**								
2012	59 482	14.7	43 574	73.3	11 247	18.9	4661	7.8
2013	59 943	14.8	46 952	78.3	8443	14.1	4548	7.6
2014	66 950	16.6	51 431	76.8	10 812	16.2	4707	7.0
2015	71 069	17.6	54 949	77.3	12 024	16.9	4096	5.8
2016	74 229	18.4	56 300	75.9	13 619	18.4	4310	5.8
2017	72 544	17.9	55 216	76.1	13 393	18.5	3935	5.4
**Specimen type**								
Skin	60 456	39.7	118 737	74.0	32 092	20.0	9627	6.0
Urinary	120 309	29.8	89 029	74.0	24 062	20.0	7218	6.0
Blood	82 908	20.5	61 352	74.0	16 582	20.0	4974	6.0
Nasopharyngeal	39 557	9.8	29 272	74.0	7911	20.0	2373	6.0
All other sites	987	0.2	730	74.0	197	20.0	59	6.0

CoPS, coagulase-positive *Staphylococci*; CoNS, coagulase-negative *Staphylococci*; CVar, coagulase-variable *Staphylococci*.

Slightly over half (51.2%) of the isolates came from males. The remaining 44.8% were obtained from female patients. Similar proportions of *Staphylococcus* were reported in the different seasons of the year: autumn (25.2%), winter (25.2%), spring (25.1%) and summer (24.5%) seasons (Table 2).

Overall, the lowest number (14.7%) of *Staphylococcus* spp. was reported in 2012. However, the number increased to 18.4% by the end of 2016 and then declined to 17.9% in 2017.

**FIGURE 2:**
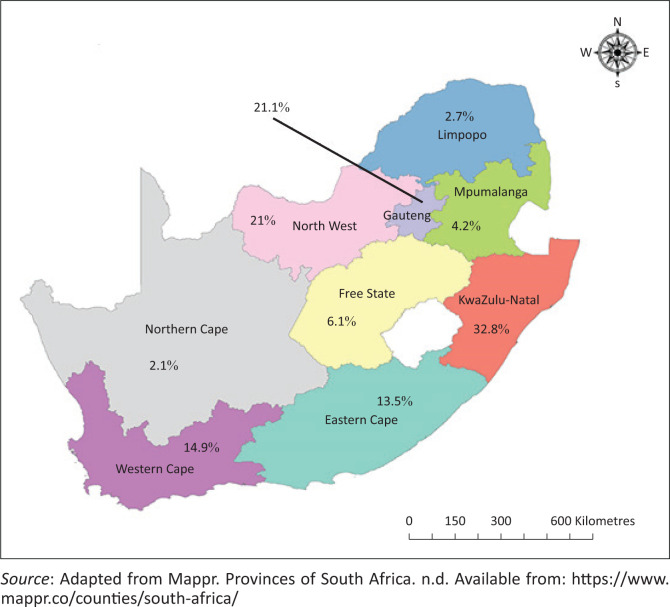
The distribution of *Staphylococcus* isolates based on the province of origin in South Africa, 2012–2017.

Figure 2 shows the number of *S. aureus, S. epidermidis, S. haemolyticus* and unspecified species reported over the study period. *Staphylococcus aureus* was the most commonly isolated throughout the study period. The number of isolates of the different species remained constant between 2012 and 2017.

## Discussion

**FIGURE 3:**
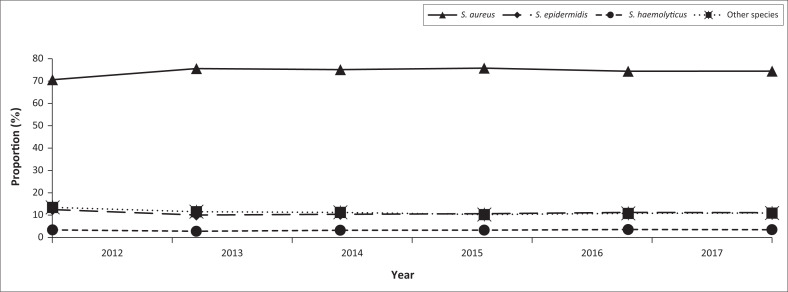
Annual patterns of the distribution of the different *Staphylococcus* spp. isolated by the NHLS in South Africa, 2012–2017.

This study describes the distribution of staphylococci isolated from human samples submitted to NHLS diagnostic laboratories. Although the CoPS group were the predominant species isolated over the study period, CoNS were also frequently isolated. *Staphylococcus aureus* was the most commonly isolated CoPS, while *S. epidermidis* and *S. haemolyticus* were the most frequently isolated CoNS. The skin samples contributed the highest number of *Staphylococcus* isolates in this study. Based on sex and age, *Staphylococcus* spp. were more frequently isolated from males than females and from children than the adults. Although the proportion of CoPS spp. observed in patients aged 0–4-year-old was lower than older age groups, a higher proportion of CoNS isolates was observed among patients aged 0–4 years than older age groups. While there was evidence of differences in the proportion of staphylococci by province in Figure 3,^16^ with KZN (KwaZulu-Natal province) NHLS (National Health Laboratory Service) contributing the highest number, there was no seasonal variation in the number of *Staphylococcus* isolated.

### Distribution of *Staphylococcus* spp. based on coagulase reaction

Among *Staphylococcus* spp. isolated in this study, the majority belonged to the CoPS group. This was expected because CoPS are commonly associated with several clinical conditions in humans. This notwithstanding, CoNS were also frequently identified. This is consistent with the findings by Martineau et al.^17^ who reported CoNS as urinary pathogens.

Among the CoPS isolated in this study, *S. aureus* was the most frequently isolated followed by *S. intermedius* and *S. pseudintermedius.* Similar findings were reported among Native Americans of the Navajo Nation,^18^ where *S. aureus* was the most common species among CoPS. This could be because of *S. aureus* being better adapted to live on the skin surface of humans than other CoPS such as *S. intermedius* and *S. pseudintermedius.*^19,20,21,22^ In addition, this difference could be explained by *S. aureus* being able to spread from person to person by direct contact, through contaminated objects or less often, by inhalation of infected droplets dispersed by sneezing or coughing. Furthermore, *S. aureus* carriers can also move the bacteria from their nose to other body parts with their hands, sometimes leading to infection.^11,23^ However, caution must be exercised in cases where *S. aureus* is encountered on the skin or mucous membrane as these organisms are frequent members of the microbiota.^24^

Among the CoNS, *S. epidermidis* and *S. haemolyticus* were more frequently isolated in this study. These two organisms can colonise the mucosal surfaces of the human nose.^25^ Other CoNS implicated in clinical conditions and observed in this study included *S. saprophyticus, S. lugdunensis, S. capitis, S. hominis* and *S. simulans.*^6,11^ Coagulase-negative staphylococci such as *Staphylococcus epidermidis*, are regarded as contaminants unless cultured from two or more independent culture specimens within a 48-h period, in which case they are then regarded as pathogens.^6^ In view of this, given that CoNS were not stratified into pathogens and contaminants we could not establish if the CoNS isolates in our study were contaminants or pathogens.

A recent study conducted in South Africa reported a 23.1% prevalence of CoNS infection in bloodstream infections among children oncology patients.^26^ However, this study is the first study to report on the spectrum of the different CoNS species isolated from patients in South Africa.

### *Staphylococcus* spp. by specimen type, gender, age, province and season

In this study, most *Staphylococcus* spp. were isolated from skin specimens. This is in contrast to findings of other studies that have reported the nasopharyngeal system of humans as the favourite site for isolation.^27,28^ The low isolation rate of *Staphylococcus* spp. from the nasopharyngeal system was therefore not expected. However, this finding is worth noting given that upper airway pathogens from the nasopharynx area are associated with lower airway infection. This is particularly important in children because the anatomy of the upper airways favours micro-aspiration of heavy bacteria-laden nasal secretions which has the potential to cause chronic lower airway infections.^29^

It is worth noting that urinary specimens accounted for 29.8% of the isolates in our study. Although the high number of isolates from the urinary tract could be because of contamination, especially among children because of the difficulty associated with collecting urine from children, this finding is nonetheless important as isolation of *staphylococci* from urine samples in the absence of bacteraemia is often considered to represent colonisation.^30^ Moreover, mortality and morbidity have been shown to be significantly associated with urinary tract infection.^31,32,33^

In this study, a higher proportion of the *Staphylococcus* spp. was from male patients. These findings were expected because a number of studies suggest that males have a higher risk of *Staphylococcus* infection than their females counterparts.^6,20,29^ In addition, the incidence of postoperative *Staphylococcus* infection is said to be higher among males than females.^34^ This phenomenon has been attributed to the fact that males tend to be poor with hand-hygiene practices compared with their female counterparts.^31,32,33,35^ However, these reports notwithstanding, while the risk of contracting staphylococci infection is higher in males, there is evidence to suggest that females have a poorer outcome following infection with *Staphylococcus* spp.^31,32,33,35^

*Staphylococcus* isolates were more frequently isolated from patients ≤ 4 years. This is consistent with several studies that show that children have a higher risk of acquiring *Staphylococcus* infections.^34,36,37,38,39^ According to Power Coombs et al., the high infection rates among the children could be attributed to their immature immune system.^40,41,42,43,44,45^

Observing a higher proprotion of CoNS spp. from younger patients (0–4 year olds) as compared with older patients is worth noting. Available evidence suggests that CoNS are the most commonly isolated pathogens in the neonatal intensive care unit. Although CoNS typically demonstrate low virulence, they are associated with morbidities in the premature infant, including chronic lung disease and adverse neurodevelopmental outcomes.^46^

However, findings of the present study failed to show an increasing burden of *Staphylococcus* infection with increasing age. This is contrary to findings reported by studies that have associated increasing age with a high prevalence of staphylococcal infections.^41,46,47,48^

Compared with other provinces, KwaZulu-Natal province contributed the highest number of isolates. Although the reasons for this observation are unclear, it is possible that the high rate of *Staphylococcus* infection in KwaZulu-Natal province in comparison to other provinces could be because of differences in socioeconomic and environmental factors.^49^ For example, a European study reported that *Staphylococcus* infection displayed a typical epidemic behaviour and had a predominantly regional distribution.^50^ As this is the first attempt at investigating the spatial distribution of *Staphylococcus* spp., in South Africa, further studies are needed to investigate the role of location in the occurrence of *Staphylococcus* spp. in South Africa.

There was no seasonal variation in the number of staphylococci isolated in the present study. This was not expected given that studies carried out elsewhere have demonstrated seasonal variation in staphylococcal infection. For example, a peak incidence of staphylococcal infection was reported during the winter months in the United States, coinciding with the peak in incidence of viral infections.^51^ Likewise, in some African countries, a peak of the incidence of staphylococcal infections has been shown to coincide with incidence of viral infections during summer.^52^

### Limitations of this study

Coagulase-negative staphylococci such as *S. epidermidis* are regarded as contaminants unless they have been cultured from two or more independent blood culture specimens within a 48-h period, in which case they are then regarded as pathogens. In view of this, given that CoNS were not stratified into pathogens and contaminants in the data used in this study, it was not possible to establish if the CoNS isolates included in this study were contaminants or as a result of infection.^27^

Furthermore, results used in this study did not include information on whether the patients were inpatients or outpatients or whether intravascular catheters or other invasive devices could be sources of infection. Nonetheless, the results of this study provide useful primary information on the burden of staphylococcal infections in patients presented to public health hospitals in South Africa.

## Conclusion

A diverse number of *Staphylococcus* spp. are associated with humans in South Africa, with *S. aureus* being the predominant species. *Staphylococcus haemolyticus* and *S. epidermidis*, both with zoonotic potential, are the predominant CoNS associated with humans. Findings reported here suggest a spatial pattern in the burden of infection. Further research is needed to confirm findings of the present study and to identify determinants of the pattern of infection observed in the study group. The data used in this study does not distinguish between contaminants and infections by CoNS species. More in-depth studies that focus on contamination versus true infection are needed. Furthermore, detailed studies that seek to link the various *Staphylococcus* spp. identified in this study to clinical outcomes are needed.
